# Supplementary material for: Polymeric nature of tandemly repeated genes enhances assembly of constitutive heterochromatin in fission yeast
Source: Commun Biol. 2023 Aug 4;6:796. doi: 10.1038/s42003-023-05154-w (PMC10403545; doi:10.1038/s42003-023-05154-w)
Supplement: Supplementary file 2 — Supplemental Material [file 42003_2023_5154_MOESM2_ESM.pdf]

Supplementary Materials: Connectivity of tandemly repeated genes enhances assembly of constitutive heterochromatin in fission yeast

Tetsuya Yamamoto,[†] Takahiro Asanuma,[‡] and Yota Murakami[‡]

†*Institute for Chemical Design and Discovery, Hokkaido University*

‡*Department of Chemistry, Faculty of Science, Hokkaido University*

Supplementary Note 1: Surface adhesion of polymers

We here discuss the essence of the adhesion of a polymer to a surface. To this end, we treat a section in a long polymer chain at the vicinity of a surface, see fig. 1**a**. The polymer section is composed of N units that are adhesive to the surface. The section is connected to the surface via a linker chain composed of N_{L} units so that the adhesive section does not diffuse away from the surface ($N \ll N_{\mathrm{L}}$). The length of both units is b. We represent the positions in the system by the distance z from the surface. Because the system is symmetric with respect to the x- and y- directions, we only analyze the motion of beads in the z-direction. Each adhesive unit can bind to the surface with the rate k_{on} when it is located in $0 < z < b$. A bound unit is unbound from the surface with the rate k_{off}.

Polymer with one adhesive unit

One can solve derive the analytic form of the binding probability p for the case of a polymer that has only one adhesive unit, $N = 1$. The distribution of the adhesive unit is

$$\psi(z) = \frac{3z}{b^2 N_{\mathrm{L}}} \mathrm{e}^{-3z^2/(2b^2 N_{\mathrm{L}})}. \tag{S1}$$

The probability with which the adhesive unit is located in the region $0 < z < b$ is

$$\Psi = \int_0^b dz\, \psi(z) = 1 - \mathrm{e}^{-3/(2N_{\mathrm{L}})} \simeq \frac{3}{2N_{\mathrm{L}}}. \tag{S2}$$

The kinetic equation of the binding and unbinding of the adhesive unit has the form

$$\frac{dp}{dt} = k_{\mathrm{on}} \frac{3}{2N_{\mathrm{L}}} (1 - p) - k_{\mathrm{off}} p. \tag{S3}$$

In the steady state, $dp/dt = 0$, the binding probability is derived as

$$p = \frac{1}{1 + \frac{2}{3}\frac{k_{\rm off}}{k_{\rm on}} N_{\rm L}}. \tag{S4}$$

Rouse dynamics simulation

We performed the Rouse dynamics simulation with which a polymer chain is modeled as beads connected by springs. The polymer chain is end-grafted to a surface and the beads are labeled as $n = 0, 2, \cdots, N + N_{\rm L}$ from the grafted bead. The equation of motion of the n-th bead (Langevin equation) has the form

$$\frac{d}{dt} z_n(t) = \frac{p_n}{m} \tag{S5}$$

$$\frac{d}{dt} p_n(t) = -\frac{\partial}{\partial z_n} U - \xi p_n(t) + f_n(t) \tag{S6}$$

with $n = 0, 2, \cdots, N + N_{\rm L}$. $p_n(t)$ is the momentum of the n-th bead and $z_n(t)$ is the position (the distance from the surface) of the n-th bead. These are functions of time t. ξ is the friction constant of a bead and m is the mass of a bead. Eq. (S5) is the definition of the momentum and eq. (S6) is the Newton's equation of motion with the potential force (the first term), the friction force (the second term), and the random force $f_n(t)$ due to the thermal fluctuation (the third term). The random force follows the Gaussian statistics with the mean and correlation

$$\langle f_n(t) \rangle = 0 \tag{S7}$$

$$\langle f_n(t) f_m(t') \rangle = 2\zeta k_{\rm B} T \delta_{mn} \delta(t - t'), \tag{S8}$$

where m and n $(= 1, 2, \cdots, N + N_{\rm L})$ are indices of monomers and ζ $(= m\xi)$ is the rescaled friction constant. In general, U includes the short-range interaction due to the connectivity of the beads, the long-range interaction due to the excluded volume between the beads,

and the interaction between the beads and the surface. In our simulation, we neglect the long-range interaction (Rouse model). The potential function U has the form

$$U = \sum_{n=0}^{N+N_{\mathrm{L}}} U_{\mathrm{s}}(z_n) + \frac{1}{2}k \sum_{n=1}^{N+N_{\mathrm{L}}} (z_n - z_{n-1})^2. \tag{S9}$$

with

$$U_{\mathrm{s}}(z) = \begin{cases} U_0 + U_0 \left(\frac{z_{\mathrm{m}}^{12}}{z^{12}} - \frac{2z_{\mathrm{m}}^{6}}{z^{6}} \right) & 0 < z < z_{\mathrm{m}} \\ 0 & z_{\mathrm{m}} < z \end{cases} \tag{S10}$$

The form of eq. (S10) is the simple Lennard-Jones potential, from which the attractive part is omitted.

We integrate eqs. (S5) and (S6) numerically by using the velocity Verlet algorithm.[1] The differential equations are approximated by using the finite difference method

$$\begin{aligned} z_n(t+\Delta t) &= z_n(t) + c_1 v(t)\Delta t + c_2 a(t)\Delta t^2 + \delta r_n && \text{(S11)} \\ v_n(t+\Delta t) &= c_0 v_n(t) + (c_1 - c_2)a(t)\Delta t + c_2 a(t+\Delta t)\Delta t + \delta v_n. && \text{(S12)} \end{aligned}$$

with

$$\begin{aligned} c_0 &= \mathrm{e}^{-\xi\Delta t} && \text{(S13)} \\ c_1 &= \frac{1}{\xi\Delta t}\left(1 - \mathrm{e}^{-\xi\Delta t}\right) && \text{(S14)} \\ c_2 &= \frac{1}{(\xi\Delta t)^2}\left(\xi\Delta t - 1 + \mathrm{e}^{-\xi\Delta t}\right). && \text{(S15)} \end{aligned}$$

$v_n(t)$ $(= p_n(t)/m)$ is the velocity and $a_n(t)$ $(= -\partial U/\partial z_n)$ is the acceleration. In the numerical calculation, the positions $z_n(t)$ are rescaled by the length scale

$$l_{\mathrm{s}}^2 = \frac{k_{\mathrm{B}}T}{m}\xi^{-2}. \tag{S16}$$

and the velocity is rescaled by the scale

$$v_{\mathrm{s}} = \xi l_{\mathrm{s}}. \tag{S17}$$

The relaxation time of a monomer is

$$\tau = \frac{m\xi}{k}. \tag{S18}$$

The random values, δr_n and δv_n, follow the Gaussian distribution

$$P(\delta r_n, \delta v_n) = \frac{1}{2\pi\sigma_z\sigma_v\sqrt{1-c_{zv}^2}} \mathrm{e}^{-\frac{1}{2(1-c_{zv}^2)}\left(\frac{\delta z_n^2}{\sigma_z^2}+\frac{\delta v_n^2}{\sigma_v^2}-2c_{zv}\frac{\delta z_n}{\sigma_z}\frac{\delta v_n}{\sigma_v}\right)} \tag{S19}$$

with

$$\sigma_z^2 = \langle \delta z_n^2 \rangle = l_{\mathrm{s}}^2 \left(2\xi\Delta t - 3 + 4\mathrm{e}^{-\xi\Delta t} - \mathrm{e}^{-2\xi\Delta t}\right) \tag{S20}$$

$$\sigma_v^2 = \langle \delta v_n^2 \rangle = v_{\mathrm{s}}^2 \left(1 - \mathrm{e}^{-2\xi\Delta t}\right) \tag{S21}$$

$$\sigma_z\sigma_v c_{zv} = \langle \delta z_n \delta v_n \rangle = l_{\mathrm{s}} v_{\mathrm{s}} \left(1 - \mathrm{e}^{-\xi\Delta t}\right)^2 \tag{S22}$$

The subchain $N_{\mathrm{L}} < n < N + N_{\mathrm{L}}$ is composed of adhesive units, while the subchain $0 < n < N_{\mathrm{L}}$ is a linker. An adhesive unit can bind to the surface with a rate k_{on} when it is located in $0 < z < b$. A bound adhesive unit can unbind from the surface with a rate k_{off}. We used the mersenne twister to generate random numbers.

The initial positions of the beads are randomly generated. If the randomly generated position z of a bead is smaller than z_{m}, it is inverted to $z_n(0) = 2z_{\mathrm{m}} - z$. The system is equilibrated for time t_{eq} and then performed the simulation for time t. The ratio p_{on} of bound units and the probability q_{on} that more than one unit is bound to the surface are extracted. This simulation was performed for M times with different initial conditions. The values of parameters used for the simulation are summarized in Table 1. Our simulations

predict the binding probability for the case of a polymer with one adhesive unit in agreement with eq. (S4), see the cyan broken line and cyan dots in fig. 2. In this case, the adhesive unit is bound to the surface at any time if and only if the binding constant is zero.

Self-consistent scheme for a very long chain

To treat the surface adhesion of polymers analytically, we consider the case in which the adhesive unit is very long so that the end effect of the section is negligible. In this case, the probability p_∞ that a unit is bound to the surface does not depend on the choice of units and more than one unit is bound to the surface at any time. The problem that we are going to solve is basically the same as ref. [2] We focus on the l-th unit in the adhesive section. We consider the case in which the units between the $1-m+1$-th and $l+n-1$-th units are not bound to the surface and the $l-m$-th and $l+n$-th units are bound to the surface, see fig. 1**b**. The distribution of the l-th unit has the form

$$\psi_{m,n}(z) = \frac{4}{\sqrt{\pi}} \frac{z^2}{(2l_{mn}^2)^{3/2}} \mathrm{e}^{-z^2/(2l_{mn}^2)} \tag{S23}$$

with

$$l_{mn}^2 = \frac{b^2}{3} \frac{mn}{m+n}. \tag{S24}$$

This case happens with the probability $p_\infty^2(1-p_\infty)^{m+n-2}$. Averaging eq. (S23) with respect to m and n leads to the form

$$\psi(z) = \sum_{n=1}^{\infty} \sum_{m=1}^{\infty} p_\infty^2 (1-p_\infty)^{m+n-2} \psi_{m,n}(z). \tag{S25}$$

To derive eq. (S25), we assumed that $(1-p_\infty)^{m+n-2}$ decreases fast enough so that one can replace the upper bound of the sums with respect to m and n to the infinity.

The kinetics equation of the binding and unbinding of the l-th unit has the form

$$\frac{dp_\infty}{dt} = k_{\rm on}\Psi(p_\infty)(1-p_\infty) - k_{\rm off}p_\infty, \tag{S26}$$

where Ψ is the probability with which the l-th unit is located in $0 < z < b$ and has the form

$$\Psi(p_\infty) = \int_0^b dz\, \psi(z). \tag{S27}$$

By solving eq. (S26) for the steady state, $dp_\infty/dt = 0$, the binding probability p_∞ is derived as

$$\frac{k_{\rm off}}{k_{\rm on}} = \frac{(1-p_\infty)}{p_\infty}\Psi(p_\infty), \tag{S28}$$

see the black broken line in fig. 3. The right side is a function only of the binding probability p_∞ and the left side $k_{\rm off}/k_{\rm on}$ is the binding constant. Eq. (S28) agrees with the prediction of the Rouse dynamics simulation for cases in which the number N of adhesive units is large and the binding constant $k_{\rm off}/k_{\rm on}$ is small, see the dots and the black broken line in fig. 3.

Extension to adhesive section of finite length

Eq. (S28) is simpler than the Rouse dynamics simulations, but its applicability is limited, see fig. 3. Eq. (S28) is derived by assuming that the number of units in the adhesive section is infinite, while the number of units in the adhesive section is finite in the Rouse dynamics simulation. The upper bound of the number of units bound to the surface and the effect of the ends of the adhesive section are neglected in the self-consistent scheme. We here take into account the upper bound of the number of bound units in an extension of the infinite approximation.

To take into account the upper bound of the number of bound adhesive units, we analyze the kinetics of the state transition, where the state is determined by the number n of adhesive

units, see fig. 4. For cases in which at least one unit is bound to the surface, $n > 1$, the number of bound units increases with the rate $\tilde{k}_{\rm on}$ and decreases with the rate $\tilde{k}_{\rm off}$. In general, the rates $\tilde{k}_{\rm on}$ and $\tilde{k}_{\rm off}$ are different from the rates $k_{\rm on}$ and $k_{\rm off}$ because of the connectivity of the units. The rates $\tilde{k}_{\rm on}$ and $\tilde{k}_{\rm off}$ do not depend on the choice of units because we still neglect the end effect. The kinetics of the probability P_n that n units are bound to the surface has the form

$$\begin{aligned} \frac{d}{dt}P_n(t) &= (N-(n-1))\tilde{k}_{\rm on}P_{n-1} + \tilde{k}_{\rm off}(n+1)P_{n+1} \\ &\quad -(N-n)\tilde{k}_{\rm on}P_n - \tilde{k}_{\rm off}nP_n \end{aligned} \tag{S29}$$

for $2 < n < N-1$ and

$$\frac{d}{dt}P_N(t) = \tilde{k}_{\rm on}P_{N-1} - \tilde{k}_{\rm off}NP_N \tag{S30}$$

for $n = N$ because of the upper bound of the number of bound units. In the steady state, $dP_n/dt = 0$, the probability P_n has the form

$$P_n = \frac{(N-1)!}{n!(N-n)!}\left(\frac{p_\infty}{1-p_\infty}\right)^{n-1}P_1 \tag{S31}$$

for $n \leq 1$. We used

$$p_\infty = \frac{\tilde{k}_{\rm on}}{\tilde{k}_{\rm on}+\tilde{k}_{\rm off}} \tag{S32}$$

in the expression of eq. (S31) (many readers would agree that eq. (S32) is apparent, but if one wants to prove it only by mathematical operations, one should do the following calculation without using eq. (S32) and find p_∞ by taking the limit $N \to \infty$ to eq. (S35)).

The probability $q_{\rm on}$ that at least one unit is bound to the surface has the form

$$\begin{aligned} q_{\rm on} &= \sum_{n=1}^{N} P_n \\ &= \frac{P_1}{N}\frac{1-p_\infty}{p_\infty}\left(\frac{1}{(1-p_\infty)^N}-1\right). \end{aligned} \tag{S33}$$

The ratio $p_{\rm on}$ of bound units has the form

$$\begin{aligned} p_{\rm on} &= \frac{1}{N}\sum_{n=1}^{N} nP_n \\ &= \frac{P_1}{N}\frac{1}{(1-p_\infty)^{N-1}} \end{aligned} \tag{S34}$$

The (conditional) probability p that an arbitrary unit is bound to the surface in the condition that at least one unit is bound to the surface thus has the form

$$p = \frac{p_{\rm on}}{q_{\rm on}} = \frac{p_\infty}{1-(1-p_\infty)^N}. \tag{S35}$$

Eq. (S35) returns to $p = p_\infty$ for $N \to \infty$ and to $p = 1$ for $N = 1$. Eq. (S35) greatly improves the infinite approximation, see the dots and solid lines in fig. 3.

Scaling theory

The right side of eq. (S28) is a complex function of p_∞. We here derive a simple approximate form of p_∞ by using the scaling argument by de Gennes.[3] The number of units bound to the surface is $p_\infty N$ and thus, on average, every p_∞^{-1} units are bound to the surface. The size of a subsection between neighboring bound units is $\xi_{\rm i} = bp_\infty^{-1/2}$ if the subsection is a ideal chain. Each unit is thus confined in a layer of thickness $\xi_{\rm i}$ at the surface, see fig. 1**c**. The kinetic equation of the binding and unbinding of units thus has the form

$$\frac{d}{dt}p_\infty = k_{\rm on}\frac{b}{\xi_{\rm i}}(1-p_\infty) - k_{\rm off}p_\infty, \tag{S36}$$

where b/ξ in the first term represents the fact that a unit is confined in a layer of thickness $\xi_{\rm i}$. In the steady state, $dp_\infty/dt = 0$, eq. (S36) has the solution

$$\frac{k_{\rm off}}{k_{\rm on}} = \frac{1 - p_\infty}{\sqrt{p_\infty}}. \tag{S37}$$

Eq. (S37) can be even solved in terms of p_∞ as

$$p_\infty = 1 + \frac{1}{2}\left(\frac{k_{\rm off}}{k_{\rm on}}\right)^2 - \sqrt{\frac{1}{4}\left(\frac{k_{\rm off}}{k_{\rm on}}\right)^4 + \left(\frac{k_{\rm off}}{k_{\rm on}}\right)^2}. \tag{S38}$$

Numerically, eq. (S37) agrees well with eq. (S28), see fig. 5. We thus use this scaling theory to take into account the polymeric nature of tandemly repeated genes in the binding of nascent RNAs to RDRC/Dicers at the surface of the nulcear envelope and use eq. (S35) to take into account the fact that the number of genes in the repeat is finite. Fig. 1**c** in the main article is derived by substituting eqs. (S38) into eq. (S35) (p is the fraction of bound units, provided that at least one unit is bound to the surface).

Binding probability

The probability $q_{\rm on}$ $(= 1 - P_0)$ that more than one unit is bound to the surface is derived by the kinetic equation

$$\frac{d}{dt}P_0 = -k_{\rm on}N\Psi P_0 + k_{\rm off}P_1, \tag{S39}$$

see fig. 4. For the case of $N \ll N_{\rm L}$, Ψ is approximated to eq. (S2. In the steady state, P_1 has the form

$$P_1 = \frac{k_{\rm on}}{k_{\rm off}}N\Psi(1 - q_{\rm on}). \tag{S40}$$

By using eq. (S33), the binding probability q_{on} has the form

$$q_{\mathrm{on}} = \frac{1}{1 + \frac{k_{\mathrm{off}}}{k_{\mathrm{on}}} \Psi^{-1} \frac{p_{\infty}(1-p_{\infty})^{N-1}}{1-(1-p_{\infty})^{N}}}. \tag{S41}$$

Fig. 1**b** in the main article is derived by substituting eq. (S38) into eq. (S41).

Supplementary Note 2: Derivation of Fig. 4

We here summarize the derivation of Fig. 4 in the main article. The kinetic equation of the binding probability p of a chromatin unit (that corresponds to p_∞ in Supplementary Note 1) has the form

$$\frac{dp}{dt} = k_{\rm on}\frac{b}{\xi}\sigma n_{\rm elo}(1-p) - \frac{p}{\tau_{\rm elo}}, \tag{S42}$$

where the first and second terms are the binding and unbinding rates of a chromatin unit, respectively, see eq. (2) in the main article. $k_{\rm on}$ is the rate constant of the binding of chromatin units. b is the Kuhn length of chromatin. ξ $(bp^{-1/2})$ is the size of the subchain of length p^{-1}. $n_{\rm elo}$ is the fraction of genes that are in the elongation state. $\tau_{\rm elo}$ is the elongation time. The fraction $n_{\rm elo}$ is given in eq. (1) in the main article, but it is convenient to rewrite it as

$$\frac{1}{k_{\rm on}\tau_{\rm elo}} = \frac{k_{\rm ini}}{k_{\rm on}}\frac{\rho}{\rho+K_{\rm p}}\frac{1-n_{\rm elo}}{n_{\rm elo}}. \tag{S43}$$

The kinetics of the degree σ of H3K9 methylation has the form

$$\frac{d\sigma}{dt} = k_{\rm m}\Lambda n_{\rm elo}(1-\sigma) - k_{\rm dm}\sigma, \tag{S44}$$

where the first and second terms are the rates of H3K9 methylation and demethylation, respectively, see eq. (5) in the main article. $k_{\rm m}$ is the rate constant that accounts for H3K9 methylation. Λ is the fraction of units to which RITS complexes are bound via small RNAs and is a function of p (see eq. (6) in the main article). $k_{\rm dm}$ is the rate constant that accounts for H3K9 demethylation.

Next, we derive the degree σ of H3K9 methylation and the binding probability p for the

steady state. The degree σ is the derived in the form

$$\sigma = \frac{n_{\mathrm{elo}}\Lambda}{n_{\mathrm{elo}}\Lambda + K_{\mathrm{m}}} \tag{S45}$$

by using eq. (S44) with $d\sigma/dt = 0$. We used the equilibrium constant

$$K_{\mathrm{m}} = \frac{k_{\mathrm{dm}}}{k_{\mathrm{m}}} \tag{S46}$$

in the form of eq. (S45). By using eqs. (S43) and (S45), eq. (S42) is rewritten in the form

$$\frac{k_{\mathrm{ini}}}{k_{\mathrm{on}}} \frac{\rho}{\rho + K_{\mathrm{p}}} \frac{1 - n_{\mathrm{elo}}}{n_{\mathrm{elo}}} = \frac{b}{\xi} \frac{1-p}{p} n_{\mathrm{elo}} \frac{n_{\mathrm{elo}}\Lambda/K_{\mathrm{m}}}{1 + n_{\mathrm{elo}}\Lambda/K_{\mathrm{m}}} \tag{S47}$$

with $dp/dt = 0$. Fig. 4 in the main article was derived by numerically solving eq. (S47) with respect to n_{elo} as a function of p and then used eq. (S43) to convert n_{elo} to $1/(k_{\mathrm{on}}\tau_{\mathrm{elo}})$.

The binding probability p at the stability limit (that gives τ_{sp1} and τ_{sp2}) is derived by the condition

$$\frac{d}{dp} n_{\mathrm{elo}} = 0, \tag{S48}$$

where the form of dn_{elo}/dp was derived by the implicit function theorem. The solutions of eq. (S48), p_{sp1} and p_{sp2}, are converted to τ_{sp1} and τ_{sp2} by using eq. (S43). Eq. (S48) has solutions only for the case of

$$\left. \frac{d}{dp} n_{\mathrm{elo}} \right|_{p=p_{\mathrm{c}}} < 0, \tag{S49}$$

where p_{c} is the value of p at the minimum of dn_{elo}/dp and is derived by the condition

$$\frac{d^2}{dp^2} n_{\mathrm{elo}} = 0. \tag{S50}$$

The number N_c of genes in the tandem repeat at the critical point was derived by the condition

$$\frac{d}{dp} n_\mathrm{elo} \bigg|_{p=p_\mathrm{c}} = 0. \tag{S51}$$

Supplementary Note 3: Derivation of eq. (9)

Free energy

The free energy of a subchain composed of g units has the form

$$F(\xi, \rho) = F_{\mathrm{ela}} + F_{\mathrm{int}} + F_{\mathrm{mix}} + F_{\mathrm{lag}}, \tag{S52}$$

which is composed of the elastic free energy F_{ela} (the first term), the mixing free energy F_{mix} (the second term), the interaction free energy F_{int} (the third term), and the Lagrange multiplier F_{lag} (the fourth term), see eq. (17) in the main article. The free energy is a function of the size ξ of the subchain and the volume fraction ρ of Pol IIs. The most stable state is given by the minimum of the free energy. ξ and ρ are derived by the conditions

$$\frac{\partial F}{\partial \rho} = 0 \tag{S53}$$

$$\frac{\partial F}{\partial \xi} = 0. \tag{S54}$$

The elastic free energy of the subchain has the form

$$\frac{F_{\mathrm{ela}}}{k_{\mathrm{B}}T} = \frac{3}{2}\frac{\xi^2}{gb^2} + \frac{3}{2}\frac{gb^2}{\xi^2}. \tag{S55}$$

Eq. (S55) results from the conformational entropy cost due to the stretching (the first term) and compression (the second term) of the subchain. ξ is the size of the subchain and b is the Kuhn length. $k_{\mathrm{B}}T$ is the thermal energy (k_{B} is the Boltzmann constant and T is the absolute temperature).

The interaction free energy has the form

$$\frac{F_{\mathrm{int}}}{k_{\mathrm{B}}T} = \frac{\xi^3}{b^3}\left[-\chi\sigma^2\left(\frac{b^3 g}{\xi^3}\right)^2 - \chi_0\left(\frac{b^3 g}{\xi^3}\right)^2\right]. \tag{S56}$$

Eq. (S56) is composed of the free energy due to the attractive interaction between the chromatin chain segments via Swi6 (the first term) and the free energy due to the excluded volume interaction between the chromatin chain segments (the second term).

The mixing free energy has the form

$$\frac{F_{\mathrm{mix}}}{k_{\mathrm{B}}T} = \frac{\xi^3}{b^3}\Bigg[\rho\log\rho + \left(1-\rho-(1+r(n_{\mathrm{on}}+n_{\mathrm{elo}}))\frac{b^3 g}{\xi^3}\right)\log\left(1-\rho-(1+r(n_{\mathrm{on}}+n_{\mathrm{elo}}))\frac{b^3 g}{\xi^3}\right)\Bigg] \quad \text{(S57)}$$

Eq. (S57) is composed of the contribution of Pol IIs (the first term) and solvent (the second term). n_{on} and n_{elo} are the fractions of genes that are in the bound and elongation states, respectively. The volume of solvent excluded by Pol IIs bound to chromatin units is taken into account by the factor $n_{\mathrm{on}} + n_{\mathrm{elo}}$. This results in the Pol II-Pol II excluded volume interaction and the Pol II-chromain excluded volume interaction. These excluded volume interactions are fully taken into account if $r = 1$ and are neglected if $r = 0$. In general, there are attractive interactions that counteract with these excluded volume interactions and are taken into account by introducing interaction parameters, analogous to χ and χ_0 in eq. (S56); this increases the number of unknown parameters and is not essential in the physics of heterochromatin assembly. For simplicity, we thus use the model with $r = 0$ in the main article. See Supplementary Figure 15 for the results of $r = 1$.

The Lagrange multiplier F_{lag} has the form

$$F_{\mathrm{lag}} = -\mu\frac{\xi^3}{b^3}\left(\rho + \frac{b^3 g}{\xi^3}(n_{\mathrm{on}}+n_{\mathrm{elo}})\right) + \Pi_{\mathrm{osm}}\xi^3, \quad \text{(S58)}$$

where μ is the chemical potential of Pol IIs and Π_{osm} is the osmotic pressure.

Pol II volume fraction

By using eq. (S55) - (S58), eq. (S53) is rewritten as

$$\frac{\partial}{\partial \rho}\left(\frac{F}{k_{\mathrm{B}}T}\right) = \frac{\xi^3}{b^3}\left[\log \rho - \log(1-\rho-\phi_{\mathrm{c}}) - \frac{\mu}{k_{\mathrm{B}}T}\right] = 0. \tag{S59}$$

By solving eq. (S59) with respect to ρ, the volume fraction ρ of Pol II is derived as

$$\rho = (1-\phi_{\mathrm{c}})\rho_0 \tag{S60}$$

with

$$\rho_0 = \frac{1}{1+\mathrm{e}^{-\mu/(k_{\mathrm{B}}T)}}. \tag{S61}$$

Chromatin volume fraction

The derivatives of each free energy contribution, eq. (S55) - (S58), is calculated as

$$\frac{\partial}{\partial \xi}\left(\frac{F_{\mathrm{ela}}}{k_{\mathrm{B}}T}\right) = \frac{3\xi}{gb^2} - \frac{3gb^2}{\xi^3} \tag{S62}$$

$$\frac{\partial}{\partial \xi}\left(\frac{F_{\mathrm{mix}}}{k_{\mathrm{B}}T}\right) = \frac{3\xi^2}{b^3}\left[\rho\log\rho + (1-\rho)\log\left(1-\rho-\frac{b^3 g}{\xi^3}(1+r(n_{\mathrm{on}}+n_{\mathrm{elo}}))\right) + \frac{b^3 g}{\xi^3}(1+r(n_{\mathrm{on}}+n_{\mathrm{elo}}))\right] \tag{S63}$$

$$\frac{\partial}{\partial \xi}\left(\frac{F_{\mathrm{int}}}{k_{\mathrm{B}}T}\right) = \frac{3b^3}{\xi^4}\left[\chi g^2\sigma^2 + \chi_0 g^2\right] \tag{S64}$$

$$\frac{\partial}{\partial \xi}\left(\frac{F_{\mathrm{lag}}}{k_{\mathrm{B}}T}\right) = -\frac{\mu}{k_{\mathrm{B}}T}\frac{3\xi^2}{b^3}\rho + \frac{\Pi_{\mathrm{osm}}}{k_{\mathrm{B}}T}3\xi^2. \tag{S65}$$

Eq. (S54) is thus rewritten as

$$\frac{\partial}{\partial \xi}\left(\frac{F}{k_{\mathrm{B}}T}\right) = \frac{3\xi^2}{b^3}\left[\frac{\Pi_{\mathrm{osm}} b^3}{k_{\mathrm{B}}T} + \frac{1}{\xi^2}\left(\frac{\xi}{gb^2} - \frac{gb^2}{\xi^3}\right) + \chi\sigma^2\left(\frac{b^3 g}{\xi^3}\right)^2 + \chi_0\left(\frac{b^3 g}{\xi^3}\right)^2 + \log\left(1-\rho-\frac{b^3 g}{\xi^3}(1+r(n_{\mathrm{on}}+n_{\mathrm{elo}}))\right) + \frac{b^3 g}{\xi^3}(1+r(n_{\mathrm{on}}+n_{\mathrm{elo}}))\right] = 0 \quad (S66)$$

This leads to the form of the osmotic pressure

$$\frac{\Pi_{\mathrm{osm}} b^3}{k_{\mathrm{B}}T} = -\frac{1}{\xi^2}\left(\frac{\xi}{gb^2} - \frac{gb^2}{\xi^3}\right) - \chi\sigma^2\left(\frac{b^3 g}{\xi^3}\right)^2 - \chi_0\left(\frac{b^3 g}{\xi^3}\right)^2 - \log\left(1-\rho-\frac{b^3 g}{\xi^3}(1+r(n_{\mathrm{on}}+n_{\mathrm{elo}}))\right) - \frac{b^3 g}{\xi^3}(1+r(n_{\mathrm{on}}+n_{\mathrm{elo}})) \quad (S67)$$

Eq. (S67) is rewritten as

$$\frac{\Pi_{\mathrm{osm}} b^3}{k_{\mathrm{B}}T} = -g^{-4/3}\phi_{\mathrm{c}}^{1/3}(1+rn_{\mathrm{elo}})^{-1/3} + g^{-2/3}\phi_{\mathrm{c}}^{5/3}(1+rn_{\mathrm{elo}})^{-5/3} - (\chi\sigma^2+\chi_0)\phi_{\mathrm{c}}^2(1+rn_{\mathrm{elo}})^{-2} - \log(1-\phi_{\mathrm{c}}) - \phi_{\mathrm{c}}, \quad (S68)$$

where we neglected the factors ρ and n_{on} by assuming that $\rho \ll 1$ and $n_{\mathrm{on}} \ll 1$. In eq. (S68), we used the volume fraction of chromatin units

$$\phi_{\mathrm{c}} = \frac{b^3 g}{\xi^3}(1+rn_{\mathrm{elo}}). \quad (S69)$$

Eq. (S68) with $r = 0$ reduces to eq. (9) in the main article. Eq. (S68) takes into account the attractive interaction between chromatin units with methylated nucleosomes via Swi6 (the third term) and the binding of Pol IIs to chromatin units (the factors including n_{elo}) in an extension of the equation of state that has been widely used to predict the coil-globule transition of a polymer by changing the solvent quality.

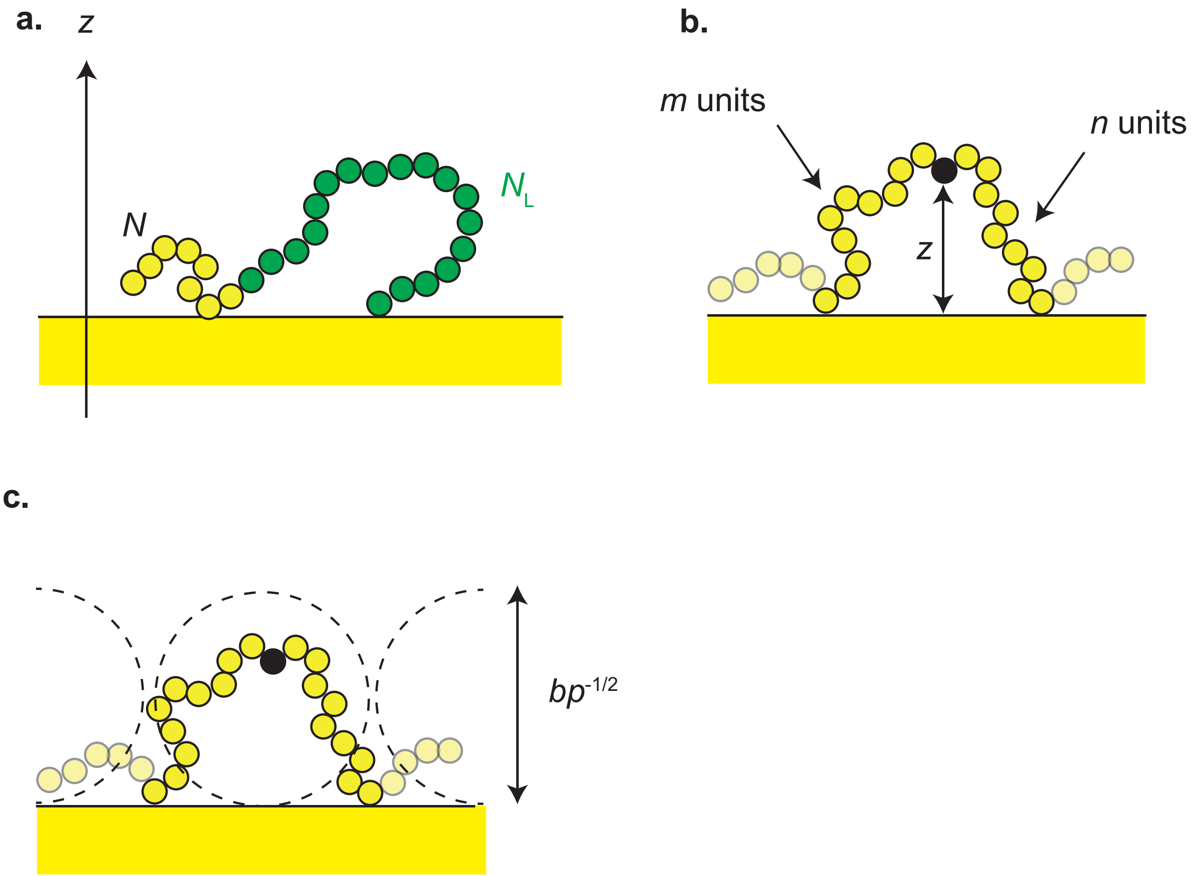

Supplementary Figure 1: **Model of polymer surface adhesion :** Models to analyze the adhesion of polymers to a surface by simulation (**a**), self-consistent scheme (**b**), and scaling theory (**c**). The adhesive units are shown by yellow beads and the linker units are shown by green beads. The positions of the beads is represented by the distance from the surface.

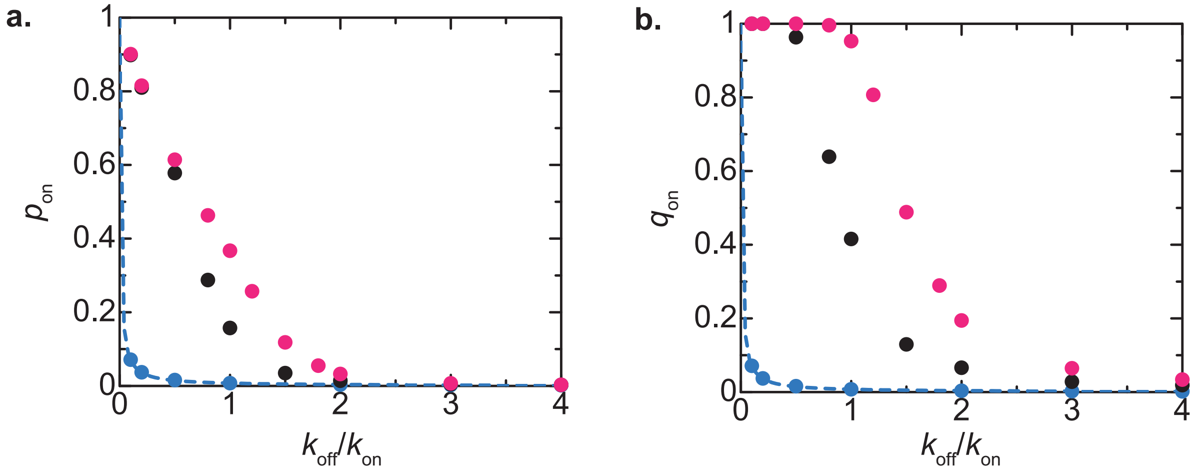

Supplementary Figure 2: **Binding probability derived by simulations:** The ratio p_{on} of bound units and the probability q_{on} that more than one adhesive unit in a polymer is bound to the surface are shown as functions of the binding constant $k_{\text{off}}/k_{\text{on}}$ for $N = 1$ (cyan), 10 (black), and 20 (magenta). The dots are derived by the Rouse dynamics simulation and the cyan broken curve is derived by using eq. (S4). The values of parameters used in the simulations are summarized in Table 1. The plotted values are the average of 5×10^4 runs.

Supplementary Table 1: Values of parameters used in the simulation.

Symbol	Meaning	Value
$\tau\xi$	Monomer relaxation time	10.0
N_{L}	Linker length	200
z_{m}/b	Width of surface-monomer interaction potential	0.3
$\xi\Delta t$	Time step	0.1
M	Number of trials	40
ξt	Simulation time	$> 4.0 \times 10^6$
ξt_{eq}	Equilibriation time	$> 4.0 \times 10^6$

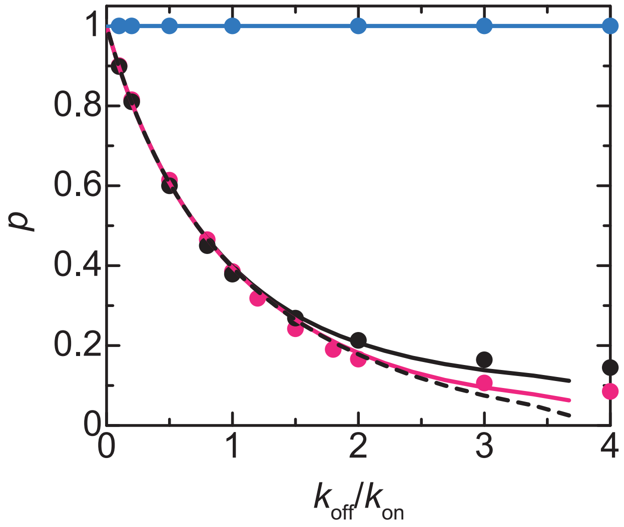

Supplementary Figure 3: **Binding probability derived by self-consistent scheme:** The conditional probability p that an arbitrary adhesive unit in a polymer to the surface, provided that at least one adhesive unit is bound to the surface, is shown as a function of the binding constant $k_{\text{off}}/k_{\text{on}}$ for $N = 1$ (cyan), 10 (black), 20 (magenta). The dots are derived by using $p = p_{\text{on}}/q_{\text{on}}$ to the data obtained by the Rouse dynamics simulation (see also fig. 2). The plotted values are the average of 5×10^4 runs. The black broken line is derived by using eq. (S28). The solid lines are derived by using eq. (S28) to eq. (S35).

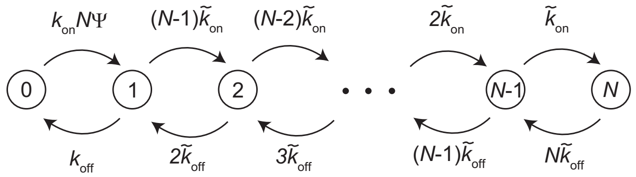

Supplementary Figure 4: **Diagram of state transitions:** We analyze the state transition to take into account the upper bound of the number of adhesive units bound to the surface. The states are represented by the number n of adhesive units bound to the surface (shown by the number in the circle). For $n > 1$, the number of adhesive units increases with the rate $\tilde{k}_{\rm on}$ and decreases with the rate $\tilde{k}_{\rm off}$. $\tilde{k}_{\rm on}$ and $\tilde{k}_{\rm off}$ are, in general, different from $k_{\rm on}$ and $k_{\rm off}$ because of the connectivity of the adhesive units and do not depend on adhesive units because we still neglect the end effect.

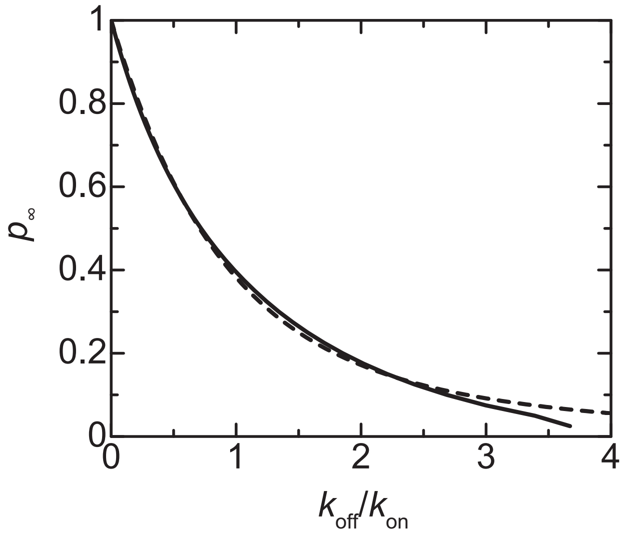

Supplementary Figure 5: **Binding probability derived by using the scaling theory:** The probability p_∞ that an arbitrary unit in a long chain is bound to the surface is shown as a function of the binding constant $k_{\text{off}}/k_{\text{on}}$. The solid line is derived by using the self-consistent scheme, see eq. (S28), and the broken line is derived by using the scaling theory, see eq. (S37).

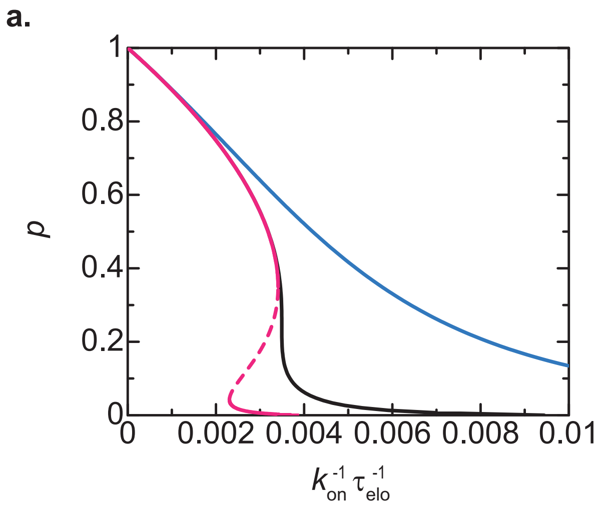

Supplementary Figure 6: **Binding probability** p **vs elongation time** τ_{elo} **with different values of methylation/demethylation rate constants** $k_{\mathrm{m}}/k_{\mathrm{dm}}$ **- ideal chain chromatin model:** The binding probability p is shown as a function of the inverse of the elongation time τ_{elo} for $s_0 k_{\mathrm{m}}/(b^2\sqrt{Dk_{\mathrm{d}}}k_{\mathrm{dm}}) = 0.01$ (**a**) and 2.0 (**b**), as predicted by using the ideal chain chromatin model. We used $N = 2$ (cyan), N_{c} (black), and 25 (magenta) for the calculations. The critical number N_{c} of genes is 9.65772 for **a** and 13.9998 for **b**. The values of other parameters used for the calculations are summarized in Table 1.

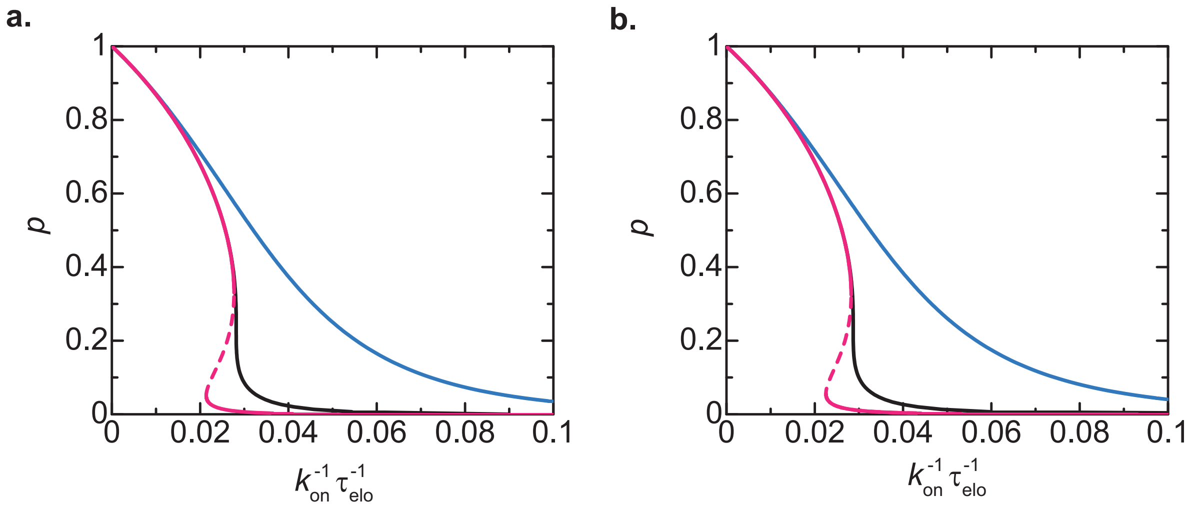

Supplementary Figure 7: **Binding probability** p **vs elongation time** $\tau_{\rm elo}$ **with different values of diffusion length** λ **- ideal chain chromatin model:** The binding probability p is shown as a function of the inverse of the elongation time $\tau_{\rm elo}$ for $\lambda/b = 20.0$ (**a**) and 50.0 (**b**), as predicted by using the ideal chain chromatin model. We used $N = 2$ (cyan), $N_{\rm c}$ (black), and 25 (magenta) for the calculations. The critical number $N_{\rm c}$ of genes is 10.6201 for **a** and 11.0376 for **b**. The values of other parameters used for the calculations are summarized in Table 1.

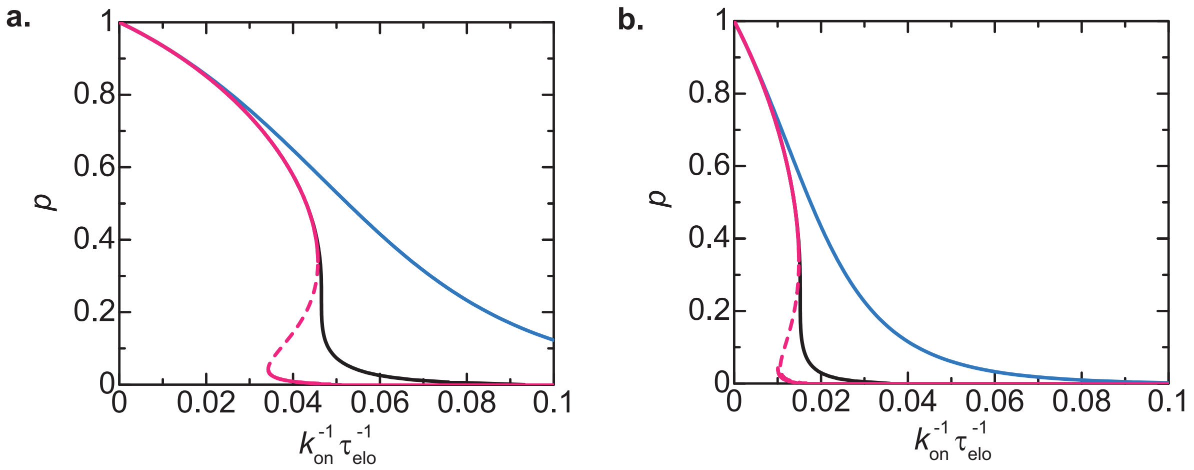

Supplementary Figure 8: Binding probability p vs elongation time τ_{elo} with different values of transcription rate constant $k_{\text{ini}}\rho_0/(\rho_0+K_{\text{p}})$ - ideal chain chromatin model: The binding probability p is shown as a function of the inverse of the elongation time τ_{elo} for $k_{\text{ini}}\rho_0/(k_{\text{on}}(\rho_0+K_{\text{p}})) = 0.1$ (**a**) and 0.4 (**b**), as predicted by using the ideal chain chromatin model. We used $N = 2$ (cyan), N_{c} (black), and 25 (magenta) for the calculations. The critical number N_{c} of genes is 9.96999 for **a** and 10.0212 for **b**. The values of other parameters used for the calculations are summarized in Table 1.

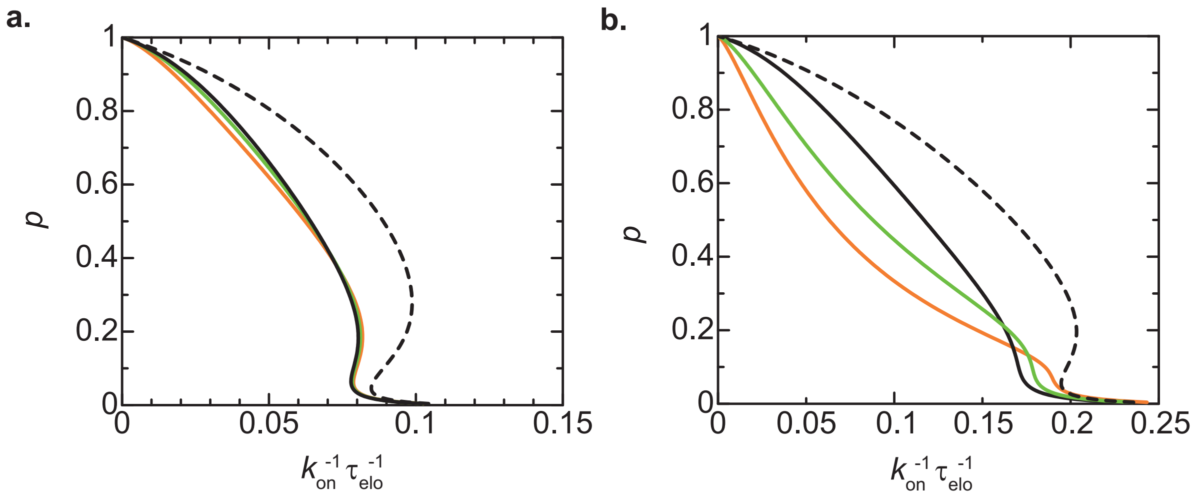

Supplementary Figure 9: **Binding probability** p **vs elongation time** $\tau_{\rm elo}$ **with different values of methylation/demethylation kinetic constants** $k_{\rm m}/k_{\rm dm}$ **- coil-globule transition model:** The binding probability p is shown as a function of the inverse of the elongation time $\tau_{\rm elo}$ for $s_0 k_{\rm m}/(b^2\sqrt{Dk_{\rm d}}k_{\rm dm}) = 1.0$ (**a**) and 5.0 (**b**), as predicted by using the coil-globule transition model (see also Figs. 6 and 7). We used $\chi = 0.0$ (black), 5.0 (light green), and 10.0 (orange) for the calculations. The black broken line is derived by using eq. (9) with $\rho = \rho_0$. The number N of units in the tandemly repeated genes is set to 25. The values of other parameters used for the calculations are summarized in Table 2.

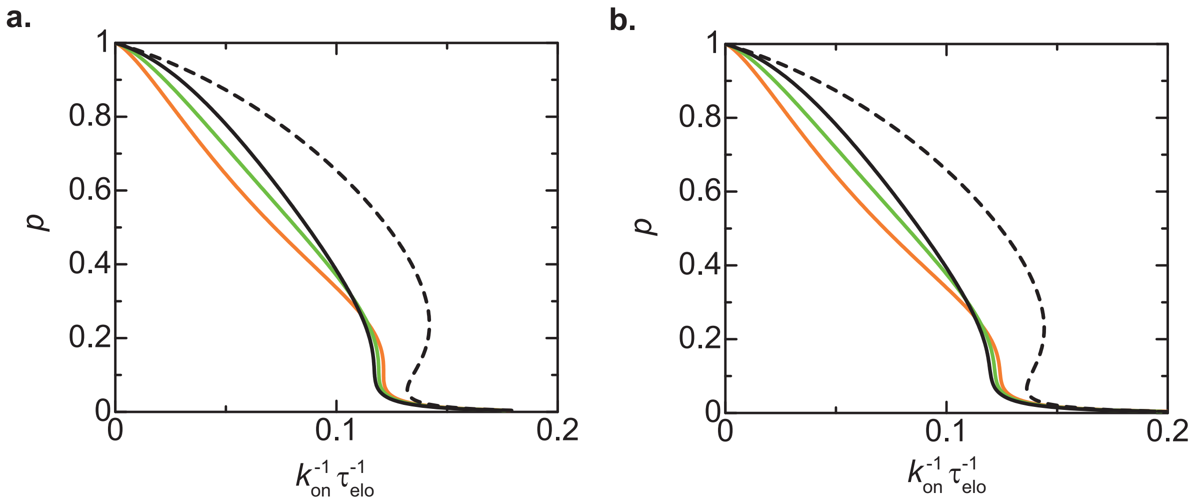

Supplementary Figure 10: **Binding probability** p **vs elongation time** $\tau_{\rm elo}$ **with different values of diffusion length** λ **- coil-globule transition model:** The binding probability p is shown as a function of the inverse of the elongation time $\tau_{\rm elo}$ for $\lambda/b = 20.0$ (**a**) and 50.0 (**b**), as predicted by using the coil-globule transition model. We used $\chi = 0.0$ (black), 5.0 (light green), and 10.0 (orange) for the calculations. The black broken line is derived by using eq. (9) with $\rho = \rho_0$. The number N of units in the tandemly repeated genes is set to 25. The values of other parameters used for the calculations are summarized in Table 2.

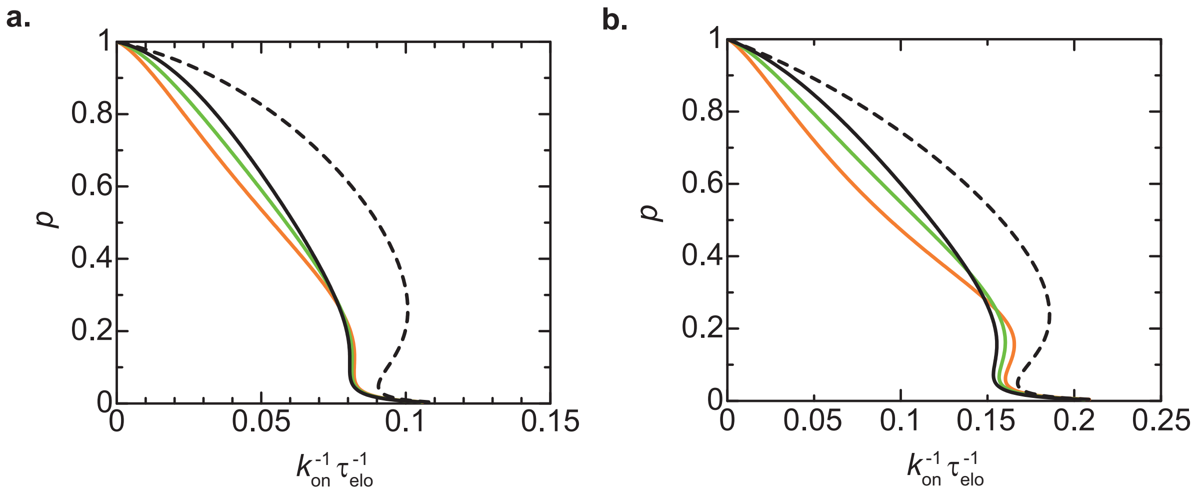

Supplementary Figure 11: **Binding probability** p **vs elongation time** τ_{elo} **with different values of transcription rate constant** k_{ini} **- coil-globule transition model:** The binding probability p is shown as a function of the inverse of the elongation time τ_{elo} for $k_{\mathrm{ini}}/(k_{\mathrm{on}}K_{\mathrm{p}}) = 0.75$ (**a**) and 3.0 (**b**), as predicted by using the coil-globule transition model. We used $\chi = 0.0$ (black), 5.0 (light green), and 10.0 (orange) for the calculations. The black broken line is derived by using eq. (9) with $\rho = \rho_0$. The number N of units in the tandemly repeated genes is set to 25. The values of other parameters used for the calculations are summarized in Table 2.

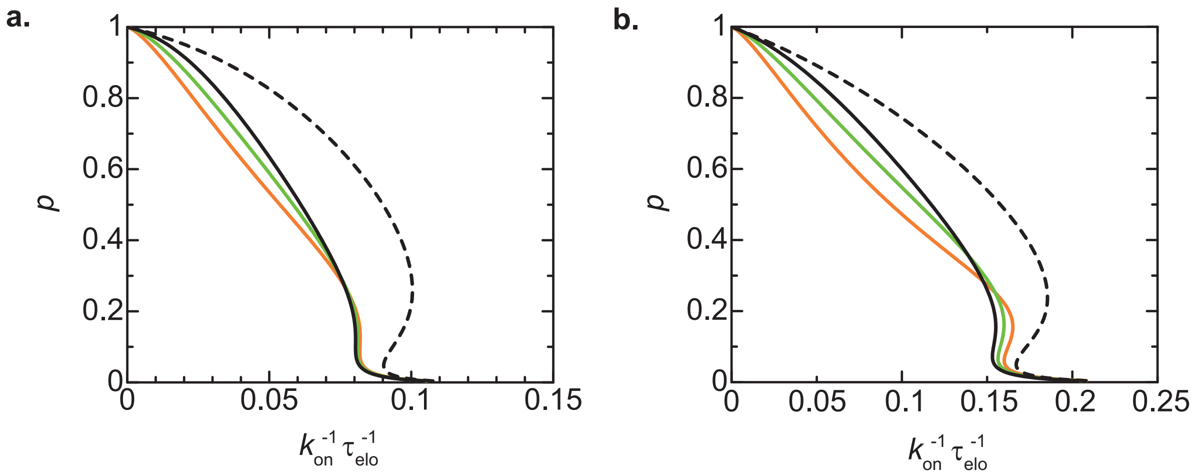

Supplementary Figure 12: **Binding probability** p **vs elongation time** τ_{elo} **with different values of Pol II volume fraction** ρ_0 **- coil-globule transition model:** The binding probability p is shown as a function of the inverse of the elongation time τ_{elo} for $\rho_0 = 0.06$ (**a**) and 0.24 (**b**), as predicted by using the coil-globule transition model. We used $\chi = 0.0$ (black), 5.0 (light green), and 10.0 (orange) for the calculations. The black broken line is derived by using eq. (9) with $\rho = \rho_0$. The number N of units in the tandemly repeated genes is set to 25. The values of other parameters used for the calculations are summarized in Table 2.

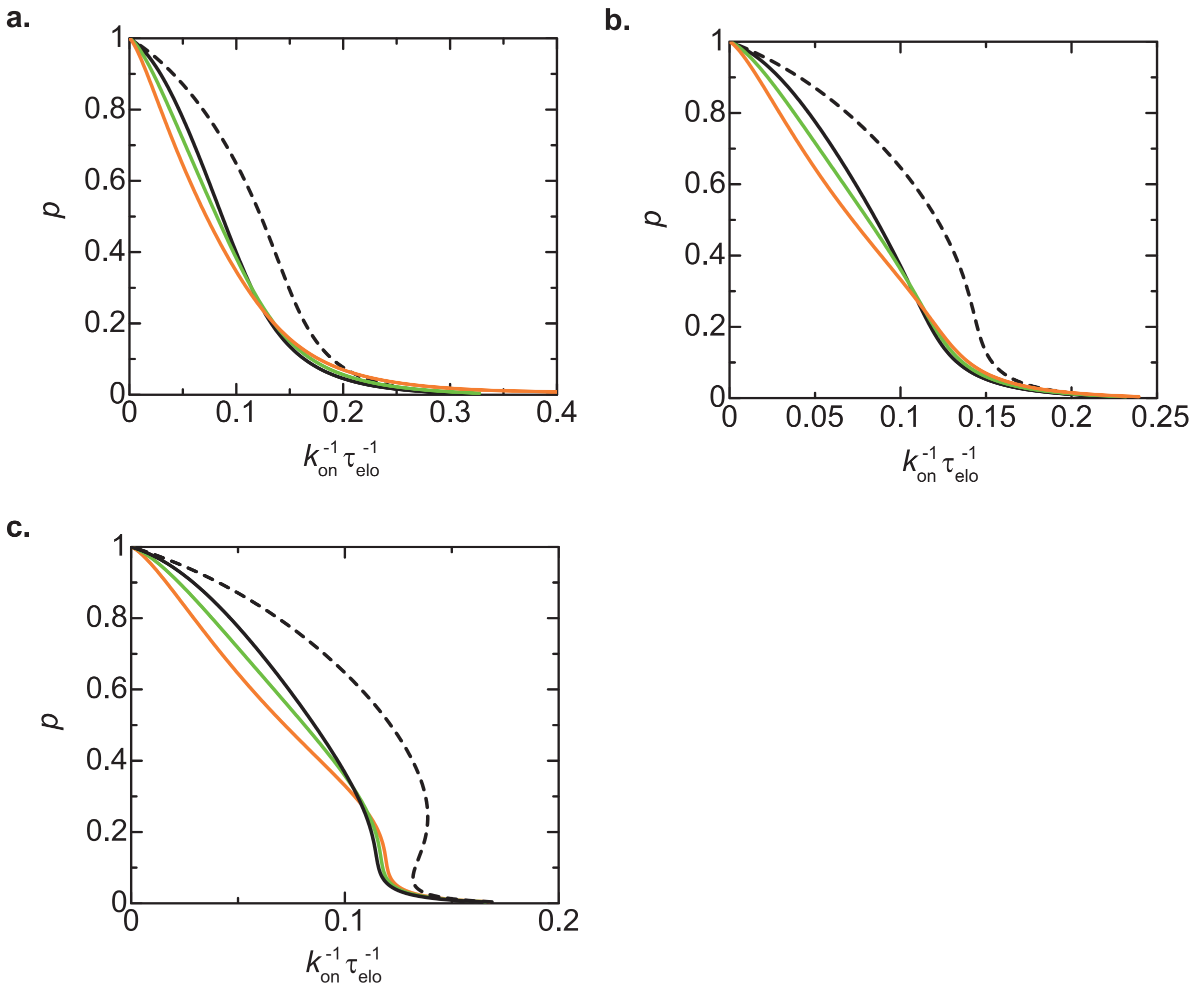

Supplementary Figure 13: **Binding probability** p **vs elongation time** $\tau_{\rm elo}$ **with different values of number** N **of genes in the tandem repeat - coil-globule transition model:** The binding probability p is shown as a function of the inverse of the elongation time $\tau_{\rm elo}$ for $N = 5$ (**a**), 10 (**b**), and 20 (**c**) as predicted by using the coil-globule transition model. We used $\chi = 0.0$ (black), 5.0 (light green), and 10.0 (orange) for the calculations. The black broken line is derived by using eq. (9) with $\rho = \rho_0$. The values of other parameters used for the calculations are summarized in Table 2.

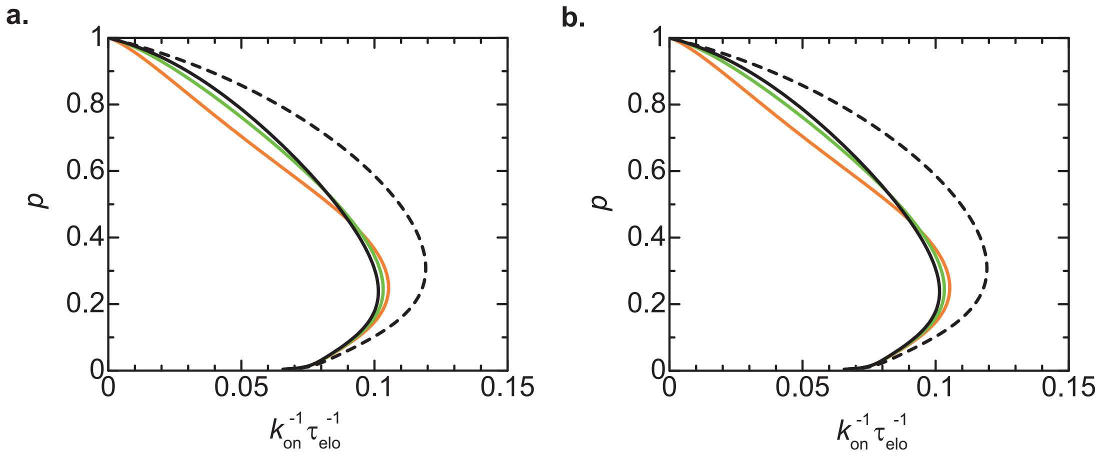

Supplementary Figure 14: **Binding probability** p **vs elongation time** τ_{elo} **with different values of interaction parameter** χ_0 **- coil-globule transition model:** The binding probability p is shown as a function of the inverse of the elongation time τ_{elo} for $\chi_0 = 0.0$ (**a**) and 0.25 (**b**), as predicted by using the coil-globule transition model. We used $\chi = 0.0$ (black), 5.0 (light green), and 10.0 (orange) for the calculations. The black broken line is derived by using eq. (9) with $\rho = \rho_0$. The values of other parameters used for the calculations are summarized in Table 2.

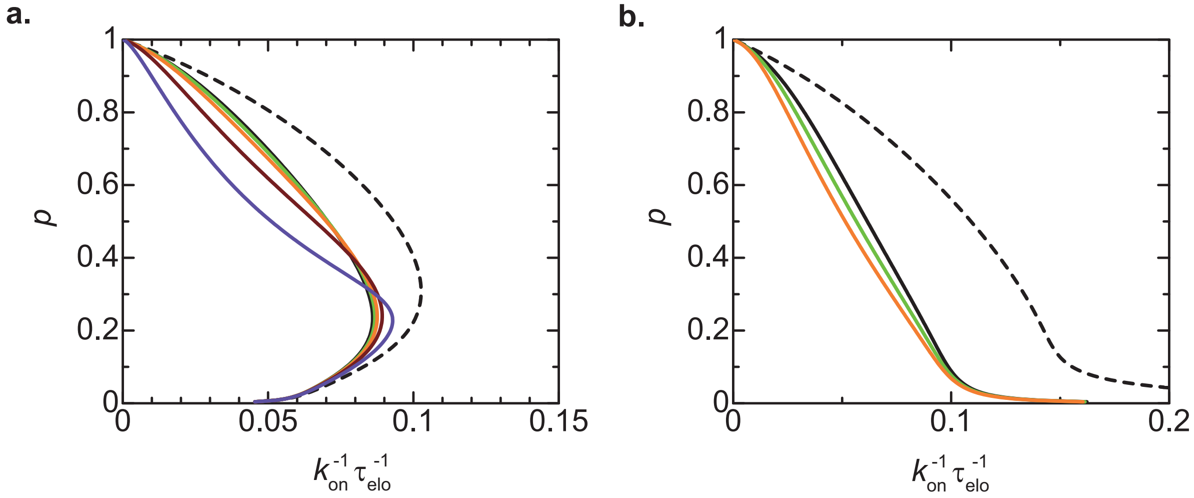

Supplementary Figure 15: **Binding probability** p **vs elongation time** $\tau_{\rm elo}$ **- coil-globule transition model with** $r = 1$**:** The binding probability p is shown as a function of the inverse of the elongation time $\tau_{\rm elo}$ for $\chi_0 = 0.5$ (**a**) and 2.0 (**b**), as predicted by using the coil-globule transition model with $r = 1$. We used $\chi = 0.0$ (black), 5.0 (light green), 10.0 (orange), 20.0 (brown), and 40.0 (violet) for the calculations. The cases of $\chi = 20.0$ and 40.0 are shown only in **a**. The black broken line is derived by using eq. (9) with $\rho = \rho_0$. The values of other parameters used for the calculations are summarized in Table 2.

Supplementary References

(1) Allen MP and Tildesley DJ (2017) Computer Simulation of Liquids, 2nd ed., Oxford Univ. Press. Oxford. UK.

(2) Silberberg A (1962) The adhesion of flexible macromolecules. Part 1. The isolated macromolecule at a plane interface. J. Phys. Chem., 66: 1872-1883.

(3) de Gennes PG (1979) Scaling concepts in polymer physics. Cornell Univ. Press. NY. USA.

Supplementary Table 2: Glossary of symbols used in the main article.

Symbol	Meaning	Eq.
q_{on}	Probability of bound state (more than one unit bound to RDRC/Dicers)	7
p	Probability of chromatin units binding to RDRC/Dicers (at bound state)	2
σ	Degree of H3K9 methylation	5
n_{elo}	Fraction of genes in elongation state	1
ϕ_{c}	Chromatin volume fraction	9
ξ	Size of subchain composed of p^{-1} units	8,9
ρ	Pol II volume fraction	10
S	Small RNA production rate	3
$c(z)$	Local volume fraction of small RNAs	4
ξ_N^2	Area occupied by tandemly repeated genes	3
λ	Diffusion length of small RNAs	4
Λ	Probability: RITS-small RNA complex binding to nascent RNA	6
b	Length of a chromatin unit (= a gene)	
N	Number of units (genes) in tandemly repeated genes	
K_{p}	Equilibrium constant: Pol II binding/unbinding to promoters	
k_{ini}	Rate constant: transcription initiation	
τ_{elo}	Elongation time	
ρ_0	Pol II volume fraction in nucleosol	
k_{on}	Rate constant: Chromatin units binding to RDRC/Dicers	
s_0	Small RNA production rate from a gene	
k_{d}	Rate constant: Small RNA degradation	
D	Small RNA diffusivity	
k_{m}	Rate constant: H3K9 methylation	
k_{dm}	Rate constant: H3K9 demethylation	
N_{L}	Number of units in linker DNA	
σ_0	Degree of H3K9 methylation due to primary RNAs	
χ	Chromatin-chromatin interaction parameter via Swi6	
χ_0	Chromatin-chromatin interaction parameter	
n_{on}	Fraction of genes in Pol II-bound state	13
n_{off}	Fraction of genes in Pol II-unbound state	14
k_{on}^{p}	Rate constant: Pol II binding to promoter	
$k_{\text{off}}^{\text{p}}$	Rate constant: Pol II unbinding from promoter	
n_0	Fraction of genes in either bound or unbound states	
F	Free energy (subchain of g units)	17
F_{ela}	Elastic free energy (subchain of g units)	18
F_{mix}	Mixing free energy (subchain of g units)	19
F_{int}	Interaction free energy (subchain of g units)	20
μ	Chemical potential of Pol II	17
Π_{osm}	Osmotic pressure	17
z	Distance from nuclear membrane surface	
